# Generic care pathway for elderly patients in need of home care services after discharge from hospital: a cluster randomised controlled trial

**DOI:** 10.1186/s12913-017-2206-3

**Published:** 2017-04-17

**Authors:** Tove Røsstad, Øyvind Salvesen, Aslak Steinsbekk, Anders Grimsmo, Olav Sletvold, Helge Garåsen

**Affiliations:** 1 Department of Public Health and Nursing, Norwegian University of Science and Technology (NTNU), Trondheim, Norway; 2 Department of Health and Welfare Services, Trondheim, Norway; 3 Norwegian Health Net, Trondheim, Norway; 4 Department of Geriatrics, St. Olavs Hospital, Trondheim University Hospital, Trondheim, Norway

**Keywords:** Health service research, Controlled randomised trial, Complex intervention, Care pathway, Continuity of care, Care coordination, Primary health care, Elderly, Checklists

## Abstract

**Background:**

Improved discharge arrangements and targeted post-discharge follow-up can reduce the risk of adverse events after hospital discharge for elderly patients. Although more care is to shift from specialist to primary care, there are few studies on post-discharge interventions run by primary care. A generic care pathway, Patient Trajectory for Home-dwelling elders (PaTH) including discharge arrangements and follow-up by primary care, was developed and introduced in Central Norway Region in 2009, applying checklists at defined stages in the patient trajectory. In a previous paper, we found that PaTH had potential of improving follow-up in primary care. The aim of this study was to establish the effect of PaTH—compared to usual care—for elderly in need of home care services after discharge from hospital.

**Methods:**

We did an unblinded, cluster randomised controlled trial with 12 home care clusters. Outcomes were measured at the patient level during a 12-month follow-up period for the individual patient and analysed applying linear and logistic mixed models. Primary outcomes were readmissions within 30 days and functional level assessed by Nottingham extended ADL scale. Secondary outcomes were number and length of inpatient hospital care and nursing home care, days at home, consultations with the general practitioners (GPs), mortality and health related quality of life (SF-36).

**Results:**

One-hundred and sixty-three patients were included in the PaTH group (six clusters), and 141 patients received care as usual (six clusters). We found no statistically significant differences between the groups for primary and secondary outcomes except for more consultations with the GPs in PaTH group (*p* = 0.04). Adherence to the intervention was insufficient as only 36% of the patients in the intervention group were assessed by at least three of the four main checklists in PaTH, but this improved over time.

**Conclusions:**

Lack of adherence to PaTH rendered the study inconclusive regarding the elderly’s functional level, number of readmissions after hospital discharge, and health care utilisation except for more consultations with the GPs. A targeted exploration of prerequisites for implementation is recommended in the pre-trial phase of complex intervention studies.

**Trial registration:**

Clinical Trials.gov NCT01107119, retrospectively registered 2010.04.18.

**Electronic supplementary material:**

The online version of this article (doi:10.1186/s12913-017-2206-3) contains supplementary material, which is available to authorized users.

## Background

For elderly patients characterised by multimorbidity, functional decline and complex medical regimens, the transition between general hospitals and primary care is associated with a risk of adverse events; especially concerning medication discrepancies [1, 2], insufficient information transfer [2, 3] and inadequate follow-up in primary care [2]. Three systematic reviews from 2012 to 2014 [4–6] found that several types of interventions may improve transition across care settings; multicomponent interventions incorporating both pre-discharge and post-discharge interventions seems to be most effective in reducing post-discharge adverse events [5]. Although there is broad consensus that more care must shift from hospital to primary care [7, 8], only few of the papers included in the reviews were studies of post-discharge intervention performed by primary care. However, two Scandinavian studies have shown that post-discharge interventions run by primary care can reduce readmissions [9, 10], dependence on municipal care [10] and mortality [11].

**Fig. 1:**
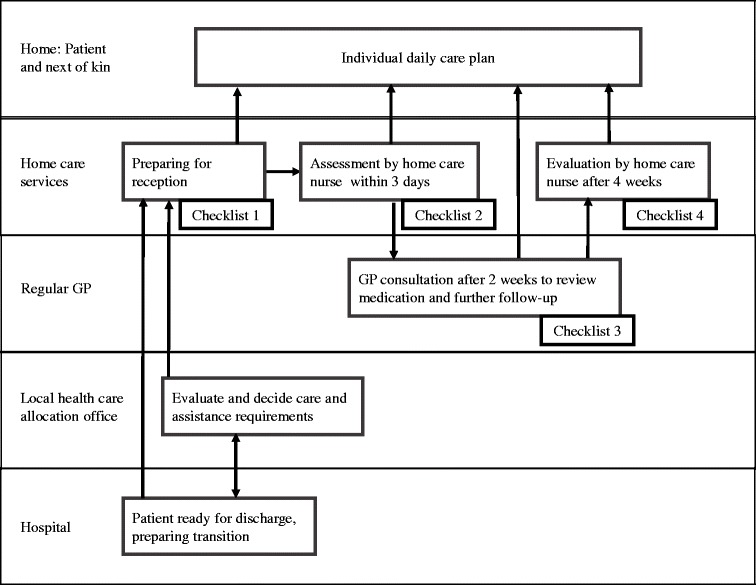
Patient trajectory for home-dwelling elders (PaTH) (12). The boxes represent procedures and checklists and the arrows the flow of information between the involved parties. The most important information from all checklists was included in the individual daily care plan which was available to all home care professionals at the point of care

The care pathway ‘Patient Trajectory for Home-dwelling elders’ (PaTH) was developed [12] and introduced [13] in Central Norway region in 2009 to ensure adequate pre-discharge planning and coordination between general hospitals and primary care for elderly patients in need of home care services after hospital discharge. Furthermore, post-discharge follow-up by home care professionals and general practitioners (GPs) was structured to ensure adequate care of medical conditions, prevent functional decline and ensure sufficient social support by introducing checklists at defined stages in the patient trajectory (Fig. 1). PaTH was developed by health care professionals from six municipalities and three hospitals who decided on a generic care pathway—suitable for patients with most diagnoses—in contrast to care pathways developed and used in hospitals targeting a defined group of patients with a specific medical condition [14].

During a qualitative process evaluation of the implementation of PaTH [13], home care professionals expressed that they were better prepared before discharge and experienced improved collaboration and exchange of information with the GPs. They also reported that the systematised observations and measures provided by using PaTH resulted in services of higher quality. The home care leaders valued PaTH as a management tool that served to facilitate change in coordination and provision of health care. These effects on the process level became gradually more apparent with time in the municipalities where the home care professionals found a way to incorporate PaTH into daily working routines.

The aim of this study was to establish the effect of PaTH on patient level—compared to usual care − for elderly patients in need of home care services after discharge from a general hospital, regarding primarily the patients’ functional level and readmissions, secondarily use of health care services, mortality and quality of life.

## Methods

This study of the effect of the recently developed PaTH [12], had an unblinded, cluster randomised controlled trial (cRCT) design and enrolled patients consecutively in the period October 2009–March 2011. It was registered in Clinical Trials.gov (NCT01107119).

### Participants

**Fig. 2:**
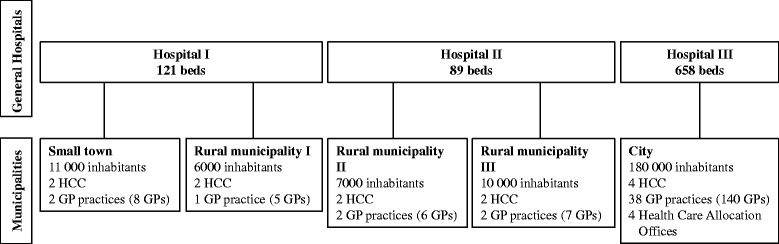
Organisation of health care services participating in PaTH. HCC = home care cluster GP = general practitioner. All hospitals serve as general hospitals to the participating municipalities. Hospital III also has regional and university functions. Every municipality has one or more home care units with nurses and nursing assistants providing health and social care to inhabitants with reduced functional level. One home care cluster included one to three home care units in the same municipality. GPs usually work in group practices and operate independently of the home care services. Every inhabitant has a right of free choice of a regular GP, which implies that the GP may have patients in common with all home care units in the municipality. Larger municipalities have health care allocation offices with municipal case managers who do a broad assessment of patients in need of municipal health and social care services other than private physiotherapy and GP services. They have a purchaser role deciding on what kind of services to be provided

The six municipalities that were involved in developing PaTH [12] were eligible to participate in this trial, and five of the local municipal authorities agreed to participate. The home care units in those five municipalities formed all together 12 home care clusters, two to four in each municipality (Fig. 2).

Patients eligible for study inclusion had to be 70 years or older and served by one of the included clusters or scheduled to receive home care services after discharge from hospital—either directly to their own homes or via an intermediate stay (anticipated duration ≤ 4 weeks) at a local rehabilitation facility or nursing home. Being served by home care services implied functional and / or cognitive impairments. However, patients were not included if their caregivers or health personnel responsible for the care services considered them to be unable to understand and sign a written consent form due to cognitive impairments.

### Randomisation and recruitment

A randomisation procedure was designed for intervention (PaTH) - and control clusters (equal numbers) from each of the participating municipalities, and an independent organisation (the Ministry of Health) performed the randomisation by drawing lots. Patients were enrolled within the randomised clusters, either by municipal case managers (Fig. 2) in discharge meetings at the hospital (city only), or by nurses in the home care services immediately after returning home. When municipal case managers recruited patients, they informed the home care professionals about the inclusion through annotations in a common electronic health record.

### Intervention

PaTH, a multicomponent complex intervention, differed from usual care by introducing new procedures for communication and follow-up, using checklists at defined stages in the patient trajectory (Fig. 1, Table 1). The detailed content of the four main checklists is presented in Additional file 1. The checklists were to be used mainly within the home care services and included assessments of health issues, social conditions and physical and cognitive functioning [13]. The purpose was to ensure closer follow-up of the patients’ medical condition and functional ability by reminding health personnel to assess, communicate and act upon relevant issues. The checklists did not describe in detail how to do this as the nurses and nursing assistants should use their professional insight to decide on how to perform necessary care.

**Table 1:** Main checklists of PaTH

Time / responsible	Procedure (s) / main themes on checklists
Discharge call from hospital to home care services at the day of discharge (Checklist 1).	Predefined information was transferred to home care services with emphasis on immediate follow-up needs and medication.
Post-discharge assessments by a home care nurse within three days (Checklist 2).	Structured assessment with emphasis on health issues, preventive measures, self-care and safety issues.
Post-discharge examination by the general practitioner (GP) within 2 weeks (Checklist 3).	Structured exchange of information between home care services and GPs before and after the GP consultation. Emphasis on observations passed on by the home care professionals, review of medical situation and medication by the GP, and plan for further follow up in collaboration between the GP and the home care services.
Post-discharge assessment by a home care professional within 4 weeks (Checklist 4).	Structured assessment with emphasis on physical / cognitive functional ability, health issues, safety issues, social situation and self-care. Evaluation of whether care matches the needs of the care recipients.

The checklists were incorporated in the electronic health records of the home care services, but the home care professionals were dependent on fax or phone when communicating with the hospital staff or the GPs.

The hospitals followed usual procedure when contacting the municipalities before discharge, but the PaTH clusters required more comprehensive information, which was defined in the checklists. Control clusters followed usual procedures regarding information exchange and follow-up. This implied that post-discharge observations and assessments of health issues after discharge were not standardised and differed between the individual health care professionals. Their focus was mainly to assist the patients in activities of daily living. Furthermore, there were no regular procedures of follow-up by the GPs post-discharge.

There was a 3-month pilot period from October 2009 involving one of the intervention clusters, resulting in some minor changes in the checklists like sequence of the themes and linguistic clarifications. PaTH was gradually introduced in all municipalities from January to March 2010. The home care professionals had a 1-day introduction course by the project group. Further training and guidance was performed by head nurses or specially trained nurses in the municipalities [13]. The GPs were informed through scheduled meetings and by information handed out.

### Outcomes and data collection

Primary outcomes were number of readmissions, defined as acute unplanned admissions for any diagnosis within 30 days [15], and functional level measured by Nottingham extended ADL scale (NEADL) at baseline, 6 and 12 months. NEADL was chosen for assessment of functional ability as this is a validated tool used in several studies, in Norway and internationally, evaluating treatment of stroke [16], hip fractures [17, 18] and rehabilitation of elderly [19].

Secondary outcomes were number and length of inpatient hospital and nursing home stays, days at home, consultations (including home visits) with the GPs, use of home care services, deaths at 6 and 12 months and health-related quality of life (SF-36) [20].

Outcomes were assessed at the level of the individual home care recipient, and data were collected during a 12-month observation period for each person. All data were collected from registries and electronic health records except for NEADL and SF-36, which were completed by the patients themselves, by health personnel in the home care services or by a research assistant (city) in dialogue with the patients. The first author extracted demographics, diagnoses, patient outcomes and consumption of health care services from electronic health records of the home care services, the GPs and the hospitals.

The degree of compliance with PaTH was measured by recording all documented use of the four main checklists (Table 1, Additional file 1) in the electronic health records of the home care services.

### Sample size estimation and statistical analyses

We did not have data on normal changes in NEADL (primary outcome) in an unselected home care population during a 12-month period. We therefore estimated sample size based on a proxy, using mobility data in the IPLOS register. IPLOS is an individual based, standardised national registration system that describes patient disability and impairment based on WHO’s classification of disabilities [21]. It has been mandatory to use for all individuals receiving public nursing home care or home care services in Norway for several years [22]. We did a survey of IPLOS data of 2300 home care recipients in the city of Trondheim during a 12-month period previous to the present study and found a mean mobility level at baseline of 2.3 points (on a 1–5 scale), a standard deviation of 0.80, and a decline in mobility of 11.5%. To identify a difference in mobility level of at least 0.3, the required sample size was estimated to be 151 patients per group (with a *t*-test) − under the assumption that the PaTH and control groups were independent samples of equal size with equal standard deviation = 0.8, power = 0.90 and α = 0.05. Due to the complexity of the model, the research group asked for external statistical assistance in estimating sample size and unfortunately did not realise until analysing the data that cluster randomisation had not been taken into account.

For analysis of the effect of PaTH, we used linear mixed models (NEADL and SF-36) and logistic mixed models (health care utilisation). With linear mixed models, site (cluster) and patient-id were set as random factors. Multiple imputations were not done as this has been found to be unnecessary when performing mixed model analyses on longitudinal data [23]. With logistic mixed models, functional level at baseline, number of chronic diseases, and ‘living alone’ were used as fixed factors. Site and patient-id were set as random factors and days at risk were accounted for, except in analyses of health care utilisation at 6 and 12 months, which did not include patient-id and days at risk.

The results are presented as an intention-to-treat analysis. Three subgroup analyses were performed. The first subgroup analysis excluded patients who died before discharge, remained in nursing home > 4 weeks after discharge, did not receive home care services, or for whom no checklists in PaTH were used. In the second analysis, patients were exposed to at least two checklists and in the third analysis at least three of the four checklists.

SPSS (version 21) was used for descriptive analyses, and R (version 2.13.1) [24] was used for mixed models analyses.

The 2010 CONSORT checklist [25] has been guiding our analyses and presentation of the methods and results.

## Results

**Fig. 3:**
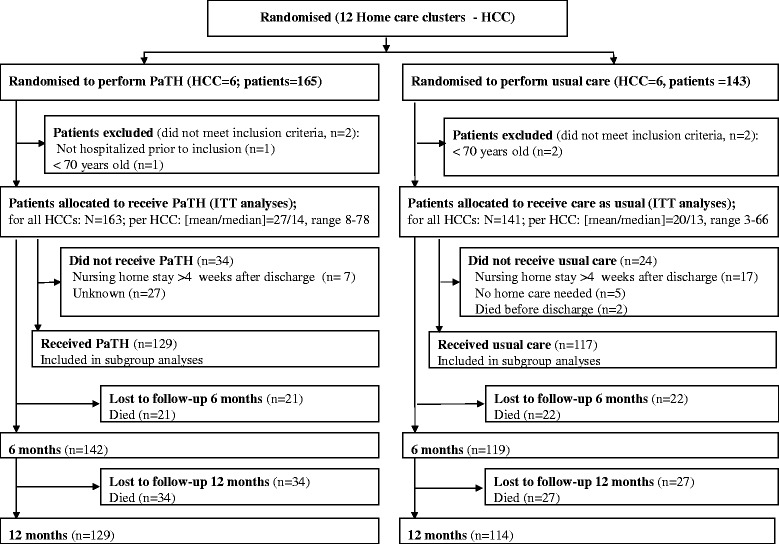
Flow of clusters and participants

Twelve home care clusters from five Norwegian municipalities included a total of 304 patients (Fig. 3). The intervention (PaTH) group and control group were comparable with respect to baseline characteristics for the patients, apart from lower functional level in the PaTH group (*p* < 0.002) (Table 2).

**Table 2:** Baseline characteristics of participants

Characteristics		PaTH group (*N* = 163)	Control group (*N* = 141)
Female sex, *n* (%)		101 (62.0%)	83 (58.9%)
Age, mean (SD), range		83.1 (5.7) 71–96	82.4 (5.7) 70–96
Living alone, *n* (%)		107 (65.6%)	97 (68.8%)
Chronic conditions^a^, mean, SD)		3.5 (2.0)	3.8 (1.8)
Primary diagnoses at index hospital stay, *n* (%)			
	Cardiac / vascular	53 (32.5%)	38 (27.0%)
	Infections	31 (19.0%)	24 (17.0%)
	Fractures / contusions	28 (17.2%)	21 (14.9%)
	Cancers	13 (8.0%)	16 (11.3%)
	Pulmonary disease	5 (3.1%)	4 (2.8%)
	Neurological disease	1 (0.6%)	8 (5.7%)
	Other diseases	32 (19.6%)	30 (21.3%)
Functional level (IPLOS score)^b^, mean (SD)		2.06 (0.47)	1.89 (0.46)

^a^Chronic diseases include established diseases like e.g. stroke, but not risk factors such as hypertension or hypercholesterolemia

^b^IPLOS data [22] consisting of 17 variables on activities of daily living, both instrumental (e.g. prepare food) and non-instrumental (e.g. personal hygiene). Lower scores imply greater independence

There were no dropouts except for deaths during the trial. Register data from hospitals and municipal care systems were complete. Printouts from the GPs’ electronic health records were missing for six patients in the PaTH group and five patients in the control group. The response rate was similar in the groups ranging from 99 to 80% for NEADL questionnaires and 59 to 76% for the SF-36 questionnaires (Table 3).

**Table 3:** Functional level (NEADL) and health related quality of life (SF-36)

Variable	Observed mean (SD) PaTH group / control group			Estimated mean difference (95% CI) / *p*-value PaTH group vs control group	
NEADL Response rate^a^	Baseline	6 months	12 months	6 months	12 months
	99% / 99%	83% / 90%	80% / 82%		
Sum score	33.3 (15.3) / 34.0 (16.0)	36.1 (17.0) / 34.9 (15.8)	35.5 (17.1) / 32.1 (16.2)	1.4 (−2.1 to 5.0) / 0.43	2.4 (−1.3 to 6.2) / 0.21
Mobility	7.4 (5.9) / 8.0 (6.1)	8.9 (6.2 / 8.4 (6.1)	8.5 (6.2) / 7.3 (6.4)	0.8 (−0.6 to 2.1) / 0.26	1.1 (−0.4 to 2.5) / 0.15
Kitchen activities	10.7 (4.5) / 10.9 (4.4)	11.4 (4.6) / 11.2 (4.6)	11.0 (4.6) / 10.8 (4.9)	0.1 (−0.9 to 1.2) / 0.79	0.01 (−1.1 to 1.2) / 0.94
Domestic activities	7.1 (4.7) / 7.2 (4.9)	7.4 (5.1) / 7.4 (4.5)	7.3 (4.9) / 6.7 (4.7)	−0.1 (−1.2 to 1.0) / 0.87	0.1 (−1.1 to 1.3) / 0.87
Leisure activities	8.2 (3.3) / 8.0 (3.7)	8.4 (4.0) / 7.9 (3.7)	8.6 (4.0) /7.5 (3.4)	0.5 (−0.4 to 1.3) / 0.26	0.6 (−0.3 to 1.5) / 0.18
SF-36 Response rate^a^	59% / 61%		76% / 72%		
PCS	30.7 (7.2) / 29.1 (8.2)		37.3 (9.6) /34.8 (10.1)		1.3 (−1.6 to 4.3) / 0.38
MCS	38.6 (9.9) / 38.0 (11.6)		46.7 (10.9) / 46.1 (12.5)		1.1 (−2.6 to 4.8) / 0.56

*Abbreviations*: *PaTH* Patient Trajectory for Home—dwelling elders, *NEADL* Nottingham extended ADL scale. The score ranges from zero to 66. Higher score implies increased independence, *SF-36* Health related quality of life, Short Form 36. Higher score implies higher quality of life, *PCS* Physical component summary, *MCS* Mental summary component

^a^Response rates of NEADL and SF-36 in percent of patients alive

All PaTH clusters used the PaTH checklists, but to varying degrees (Table 4); 79% of the patients (129 patients) were assessed by at least one, 63% (103 patients) by at least 2 and 36% (59 patients) by at least three checklists. Use of checklists improved over time; 52% of the first half of included patients in the intervention group and 75% of the last half were assessed by at least two checklists.

**Table 4:** Number of patients (%) with documented use of checklists at the PaTH sites

	Small town	Rural municipalities			City		Total
	HCC I (*n* = 17)	HCC II (*n* = 11)	HCC III (*n* = 8)	HCC IV (*n* = 8)	HCC V (*n* = 78)	HCC VI (*n* = 41)	(*n* = 163)
Adherence to PaTH							
No checklist used	0	0	0	3 (38%)	21 (27%)	10 (24%)	34 (21%)
1 checklist used	7 (41%)	0	0	2 (25%)	12 (15%)	5 (12%)	26 (16%)
2 checklists used	7 (41%)	2 (18%)	1 (13%)	1 (13%)	20 (26%)	13 (32%)	44 (27%)
3-4 checklists used	3 (18%)	9 (82%)	7 (88%)	2 (25%)	25 (32%)	13 (32%)	59 (36%)
Checklist used							
Discharge call	2 (12%)	8 (73%)	^a^	^a^	16 (21%)	23 (56%)	50^a^ (31%)
HCS assessment 3 days	17 (100%)	11 (100%)	8 (100%)	5 (63%)	53 (68%)	28 (68%)	122 (75%)
GP assessment 2 weeks	5 (29%)	8 (73%)	8 (100%)	3 (38%)	38 (49%)	16 (39%)	78 (48%)
HCS assessment 4 weeks	6 (35%)	6 (55%)	7 (88%)	2 (25%)	29 (37%)	9 (22%)	59 (36%)

*Abbreviations*: *HCC* home care cluster, *HCS* home care services, *GP* general practitioner

^a^Missing data. In rural area II and III, discharge calls were registered on paper and were not any longer available when data was collected from the electronic health records

The PaTH and control groups did not differ with respect to the primary outcomes: functional level (Table 3) and readmissions (Table 5), or the secondary outcomes: quality of life (Table 3), mortality, and health care utilisation apart from more GP consultations (*p* = 0.04) in the PaTH group (Table 5). Moreover, there were no statistical significant differences between the groups in the subgroup analyses.

**Table 5:** Health care utilisation and care situation, PaTH group vs control group

Time	Variable	PaTH (*N* = 163)	Control (*N* = 141)	Odds ratio^a^ (95%CI)	*P*-value
During 30 days	Readmissions *n* (%)	27 (16.6%)	25 (17.7%)	0.8 (0.4–1.7)	0.65
At 6 months	No care, *n* (%)	33 (20.2%)	22 (15.6%)	1.6 (0.8–3.2)	0.17
	Home care, *n* (%)	103 (63.2%)	90 (63.8%)	1.1 (0.7–1.8)	0.62
	Permanent nursing home stay, *n* (%)	6 (3.7%)	7 (5.0%)	0.4 (0.1–1.4)	0.10
	Dead, *n* (%)	21 (12.9%)	22 (15.6%)	0.7 (0.4–1.5)	0.38
At 12 months	No care, *n* (%)	30 (18.4%)	24 (17.0%)	1.0 (0.5–1.9)	0.95
	Home care, *n* (%)	86 (52.8%)	78 (55.3%)	1.1 (0.7–1.8)	0.60
	Permanent nursing home stay, *n* (%)	13 (8.0%)	12 (8.5%)	0.7 (0.3–1.7)	0.47
	Dead, *n* (%)	34 (20.9%)	27 (19.1%)	0.8 (0.4–1.6)	0.40
During 12 months	Hospital admissions (*n*) / patients (n)	244 (106)	230 (96)	1.0 (0.2–1.3)	0.77
	Days in hospital, mean (SD)	10.3 (15.0)	11.0 (15.7)	0.8 (0. 5–1.4)	0.43
	Nursing home admissions (*n*)^b^ / patients (*n*)	175 (94)	147 (85)	0.9 (0.7–1.3)	0.62
	Days in nursing homes, mean (SD)	41.4 (76.8)	45.9 (76.9)	0.7 (0.2–2.2)	0.55
	Days at home, mean (SD)	267.5 (123.7)	260.9 (127.6)	1.8 (0.9–3.4)	0.08
	GP encounters, mean (SD)^c^	5.1 (5.0)	4.4 (4.47)	1.4 (1.0–1.8)	0.04

^a^All variables are adjusted for IPLOS, number of chronic conditions and living alone. Variables measured during 12 months are accounted for days at risk

^b^Include both permanent and short term stays in nursing homes / rehabilitation facilities

^c^Available data from GPs’ electronic health records: PaTH group /control group: 157 patients (96%) / 136 patients (97%) while data on all patients were available from hospital and municipal care records

## Discussion

Lack of adherence to PaTH rendered the study inconclusive regarding the elderly’s functional level and the number of readmissions after hospital discharge. The effect of intended PaTH use could not be adequately tested because most patients (64%) were assessed by either none of the main PaTH checklists (21%) or only one or two (44%) of them (Table 4). Furthermore, we had to base the sample size estimation on a proxy as we only had access to normal decrease in ADL in the target population from the Norwegian IPLOS scoring system. The sample size estimation was not adjusted for the cluster design, which generally requires more participants than individual-controlled trials [26]. However, no cluster effects were found in this study—except for a statistical non-significant cluster effect on deaths—thus minimising this limitation.

The cluster design represented otherwise a strength of the study. This was chosen because PaTH implied a new way—involving all home care staff—to provide and organise the daily services of home care in the post-discharge period. An individual randomised design would have implied a substantial risk of contamination of the control patients, reducing the possibility of detecting effect of the intervention [27]. Other strengths are high response rate to NEADL and completeness of registry data (from hospitals and municipal care) on health care utilisation. The real-life multicentre setting is a strength, as we could test the feasibility of the intervention in several locations in a city as well as in larger and smaller rural communities. However, multicentre settings increase the complexity and can reduce the possibilities of detecting effect in a trial [28].

A promising finding in our study was a higher number of GP consultations in the PaTH group than in the control group. In two Scandinavian studies with documented effect of post-discharge interventions performed by primary care [9–11], the physicians took an active part in the medical follow-up in the early post-discharge period. In our study, the GPs and home care nurses performed independent assessments of common patients, did not share patient records, and communicated mainly by using phone or fax (Fig. 2). The PaTH procedures implied that home care staff performed the main follow-up and initiated the GP consultations, leaving the GPs in a more passive role [13]; they were merely responding to the request from the home care nurses in line with normal procedure rather than actively set the agenda for further medical follow-up as in the other Scandinavian studies [9–11]. Considering these findings, we believe that more active involvement of GPs and closer integration of follow-up by home care services and GPs in the early post-discharge phase is important to improve outcomes for this patient group characterised by multimorbidity and functional decline (Table 2).

Even if it was not possible to document other effects of PaTH on patient level in this cRCT, our former study of the implementation process [13] indicated positive effect of PaTH on the process level. Several studies have documented that care pathways can have an effect, both on the quality of health care provision and on patient level outcomes, but the effect is dependent on how the implementation process is carried out [29, 30]. A systematic review from 2012 by Smith et al. on interventions in primary care, concluded that multicomponent complex organisational interventions, such as changes in delivery of care, seem less effective on patient level outcomes than interventions directed primarily at the patients, e.g. training directed on improving activities in daily living [31]. One reason can be that implementation of complex interventions, requiring behavioural changes for the health personnel involved, takes time and require targeted efforts before the intervention is settled in the organisation [32]. This became evident in our previous implementation study [13] where we found that enthusiasm and positive attitude to the intervention was not sufficient for a successful implementation. Considerable efforts were needed to make the home care professionals understand their responsibilities in PaTH and how to assess the patients by using the checklists. A strong managerial focus on creating commitments and engagement to PaTH and practical facilitation of work processes further characterised the implementation process in the municipalities that succeeded in integrating PaTH into daily working processes.

In our cRCT, the implementation challenges were underestimated as the home care professionals had been sitting in the driver’s seat during the development process and the care pathway was developed according to their own needs of information transfer and structured follow-up [12]. Furthermore, there were no indications of implementation challenges during the pilot period. During the research period, the researcher had monthly conference calls to the contact persons in the home care services in every municipality [13]. These calls did not reveal any implementation challenges. A more targeted exploration in the pre-trial phase would have been necessary to disclose these challenges. We used the implementation theory ‘Normalisation process theory’ (NPT) [33] when exploring the implementation process [13] and see, in retrospect, that NPT could have been useful in the pilot period as well for more targeted testing of the prerequisites for implementation.

For future studies of complex interventions, we suggest, in line with the UK Medical Research Council [27], a prolonged pilot period for testing of acceptability and understanding of the intervention and furthermore, to explore the potential for implementation into daily working practices by applying an implementation theory. We further suggest that adherence to the intervention is closely recorded before preceding to the main trial to ensure that full effect can be observed within the study period.

In some of the municipalities participating in the cRCT, PATH is now in common use. Further studies are needed to evaluate the long-term sustainability as well as exploration of potential adverse events to service provision and patients.

## Conclusions

Lack of adherence to PaTH rendered the study inconclusive regarding the elderly’s functional level, number of readmissions after hospital discharge and health care utilisation except for more consultations with the GPs. A targeted exploration of prerequisites for implementation, supported by relevant implementation theories, is recommended in the pre-trial phase of complex intervention studies.

## Additional file

Additional file 1: Main PaTH checklists and individual daily care plan. (DOCX 23 kb)

## Abbreviations

CI: Confidence interval
cRCT: Cluster randomised controlled trial
GP: General practitioner
HCC: Home care cluster
HCS: Home care service
IPLOS: Individbasert pleie- og omsorgs statistikk (Individualised care statistics)
NEADL: Nottingham extended ADL scale
OR: Odds ratio
PaTH: Patient trajectory for home-dwelling elders
SF-36: Health-related quality of life, short form 36
