# Supplementary material for: Generic care pathway for elderly patients in need of home care services after discharge from hospital: a cluster randomised controlled trial
Source: BMC Health Serv Res. 2017 Apr 17;17:275. doi: 10.1186/s12913-017-2206-3 (PMC5392928; doi:10.1186/s12913-017-2206-3)
Supplement: Additional file 1: — Main PaTH checklists and individual daily care plan. (DOCX 23 kb) [file 12913_2017_2206_MOESM1_ESM.docx]

# Main PaTH checklists and individual daily care plan

### Checklist 1: Discharge call from hospital to home care services at the day of discharge.

**(Checklist to be used by the head nurse in the home care services)**

1. Name and telephone number to the ward / person calling from the hospital.
2. What kind of support is needed after discharge? Has this changed during the present hospital admission?
3. If there is a change in need for municipal health and social care, has the hospital contacted the local health care allocation office?
4. Agree on date and time for discharge.
5. Are there any specific conditions to be observed by the home care staff after discharge? This must be documented in the discharge letter.
6. Compare the list of medication used in the hospital with the list of medications in the electronic health record (EHR) of the home care services to check for new, changed or discontinued medication.
7. Make sure there is a dose-plan for warfarin the first three days.
8. Make sure that the discharge letter will be sent by fax to the home care service before 1pm at the day of discharge.
9. Make sure that the hospital provides necessary prescriptions and medications for one day / weekend.
10. If the patient has had an intermediate stay in a nursing home / rehabilitation facility, make sure that information is updated in the EHR.
11. Have necessary assistive devices been ordered? What kind of devices? Who has ordered? When will it be delivered?
12. Has the family been informed about the discharge?
13. Does the home care staff need a key for the patient’s house? Will this be delivered by the family?
14. Make sure the patient is enrolled on the work list and that services are planned according to the decision of the local health care allocation office.
15. Make sure important information is registered in the daily care plan.
16. Inform cooperating partners about the discharge.
17. Fax information about medication and immediate follow-up needs to the GP.
18. Book an appointment with the GP within 14 days if there have been changes in the medication plan during the present hospital stay or if follow-up by the GP is recommended by the hospital. Clarify whether the patient can meet at the GP practice or need a home call by the GP.
19. Make a reminder in the EHR that updated information is provided to the GP one day before the appointment.
20. Decide on time for a follow-up visit by a home care nurse.
21. Appoint a primary contact person (nurse or nursing assistant) within the home care services.

###

### Checklist 2: Post-discharge assessments by a home care nurse within three days after discharge

1. Check discharge letter from the hospital.
2. Document current acute / chronic diseases and medical conditions.
3. Check if medication list in the EHR is updated (new, changed or discontinued medication).
4. Check how medication is to be administered.
5. Observe effects / side effects of medication.
6. Remove outdated medication from the patient’s home.
7. Check that necessary assistive devices are in place in the patient’s home and that they are being used correctly.
8. Does the patient have a safety alarm? Does he understand how to use it? Does it function properly? (Test it).
9. Does the patient feel secure?
10. Does the patient cope with the situation at home?
11. Confusion about time and place?
12. Reduced short-term or long-term memory?
13. Anxiety or depressive symptoms? Paranoid symptoms?
14. Sleeping pattern.
15. Eating well? Nutritious meals? Nausea? In need of diet guidance?
16. Proper bowel function? Urinary incontinence / retention?
17. Endurance – fatigue?
18. Physical activity.
19. Ability to walk indoors and outdoors. Risk for falls?
20. Muscle strength by hand shaking.
21. Pain?
22. Signs or symptoms of infection?
23. Blood pressure (sitting and standing), pulse, circulation, respiration.
24. Make sure the recommendations in the discharge letter is being followed up.
25. What kind of conditions are especially in need of follow-up (diseases, ADL, preventive measures)? Document this in the ‘daily care plan’ in the HER.
26. Check whether there is need for other municipal health and social care services or increased home care services.
27. Update the daily care plan.
28. Plan for the four- weeks assessment together the patient’s primary contact person in the home care service.

### Checklist 3A: Procedure and information provided before examination by the GP within two weeks after discharge (home care checklist)

1. Head nurse / nurse in charge book an appointment with the GP when they are informed of the discharge.
2. Head nurse / nurse in charge clarify whether the patient can meet at the GP practice or need a home call by the GP.
3. The day before the appointment, the head nurse informs the GP in written about observations done by the home care staff concerning the patient’s health situation, physical and cognitive functional level and the patient’s ability to master daily activities.
4. This procedure is to be followed also at later consultations.

| Information from the home care services to the GP (Template in the EHR) | |
| --- | --- |
| Name of GP practice / GP:  Fax no: | |
| Name of the patient:  Date of birth / identification number: | |
| Time for appointment:  Need for a home call by the GP? (Yes / No) | |
| Current problem: | |
| Observations on physical, mental and social condition performed by home care staff:  Number of visits by home care staff per day:  Other municipal health and social care services: | |
| Contact information home care services:  Name of home care unit:  Name of head nurse:  Fax no.:  Phone: /Mobile:  Please provide feedback on conditions to be observed by the home care staff, plan for further follow-up, and advice on measures in case of exacerbations of chronic diseases |  |
| Attachment: Medication list from the EHR | |

### Checklists 3B: GP checklist for consultation two weeks after discharge

Not all issues are relevant to all patients. The GP documents as usual relevant information, but advice to home care services on further follow-up of medical condition is mandatory. The GP will receive updated information on observations and assessments from the home care professionals the day before the consultation. The GP informs at home care professionals on conclusions and plan for further follow-up after the consultation.

- Check in the discharge report or the report from the home care services whether there is a need for further examinations / test.
- Check the medication list
  - Effect or side effects of medication?
  - Can medication be reduced or simplified?
  - Has the patient understood changes in the medication list?
  - Does the patient need help to handle her / his medication?
  - Is there a need to improve the patient’s self-management related to diseases or medication?
  - Check whether there is a need for new prescriptions.
  - Make sure the medication list is updated, and information passed to the pharmacy and home care services provided they are handling the patient’s medication.
- Does the patient need rehabilitation measures?
- Agree on further follow-up consultations and inform the home care services.
- Make a follow-up plan for the home care services including
  - What to observe (e.g. cognitive functioning, effect of treatment, side effects, nutrition, weight, blood pressure, oedema. etc.).
  - How to act if the chronic medical conditions deteriorates.

### Checklist 4: Post-discharge assessment by a home care professional (nurse or nursing assistant) within four weeks after discharge.

**Health**

1. Document current acute / chronic diseases and medical conditions.
2. Document changes in patient’s health condition during the last six months.
3. Is the plan for follow-up after the last GP consultation attended to?
4. Is there a plan for further follow-up by the GP?
5. Eating well? Nutritious meals? Nausea? In need of diet guidance? Weight loss last six months? In need of help during meal?
6. Dental health: Own teeth? How is the oral hygiene? In need of a dentist?
7. Pain?
8. Proper bowel function? Urinary incontinence / retention?
9. Vision and hearing.

**Ability to make decisions on daily activities**

1. Reduced short-term or long-term memory?
2. Confusion about time and place?
3. How is the patient’s ability to plan and follow up daily activities?
4. Anxiety or depressive symptoms? Paranoid symptoms?
5. Does the patient have competence to consent to treatment?
6. Are the home conditions satisfactory (security, health, functional level)?
7. Does the patient have a safety alarm? Does he understand how to use it? Does it function properly? (Test it).
8. Does the patient feel secure?
9. Does the patient cope with the situation at home?

**Social network and activities**

1. Does she / he get any help from family / friends?
2. What kind of social activities did she / he use to attend earlier?
3. Does she / he need any help for participating in social activities?
4. Provide oral and written information about local social activities.

**Activities of Daily Living (ADL)**

1. Ability to walk indoors (walking to and from a chair, bed, toilette and staircase) and outdoors.
2. Any falls last four weeks? In need of fall-preventing measures?
3. Needs for improvements in the house due to functional impairment (e.g. remove carpets and doorsteps, better lighting)?
4. Sufficient assistive devices? Check whether they are being used correctly.
5. Personal hygiene? What can he manage himself? Need of support?
6. In need of help to dress / undress?
7. Able to prepare meals, wash dishes and cloths, do shopping, get rid of trash?
8. Able to clean his flat? If no, who is helping him?
9. Can something be done to strengthen the patient’s ADL / coping of daily activities?
10. Is there anything that the patient wants to master, but is not able to do?
11. Can something be done to reverse functional decline?
12. How is the patient’s motivation for exercising or social activities?
13. Check whether there is need of other municipal health and social care services or increased home care services.
14. Update daily care plan.

### Individual daily care plan

The most important information from all checklists is included in the individual care plan, which is updated daily. The daily care plan included the following issues:

- Time for assistance
- Relevant medical conditions
- Medication
- Observations recommended by the hospital or GP
- What to do if the condition deteriorates
- What can the home care recipient manage himself
- Preventive measures
- Assistance given by the home care professionals
